# The ‘Be Cancer Alert Campaign’: protocol to evaluate a mass media campaign to raise awareness about breast and colorectal cancer in Malaysia

**DOI:** 10.1186/s12885-018-4769-8

**Published:** 2018-09-10

**Authors:** Désirée Schliemann, Michael Donnelly, Maznah Dahlui, Siew Yim Loh, Nor Saleha Binti Tamin Ibrahim, Saunthari Somasundaram, Conan Donnelly, Tin Tin Su

**Affiliations:** 1 Centre for Public Health and UKCRC Centre of Excellence for Public Health, Queen’s, University Belfast, Belfast, UK; 2 Centre for Population Health (CePH), Department of Social and Preventive Medicine, University of Malaya, Kuala Lumpur, Malaysia; 3 Ministry of Health, Putrajaya, Malaysia; 4 National Cancer Society, Kuala Lumpur, Malaysia; 5 South East Asia Community Observatory (SEACO), Monash University Malaysia, Bandar Sunway, Malaysia; 6 National Cancer Registry Ireland, Cork, Ireland

**Keywords:** Cancer awareness, Mass media, Early detection, Malaysia, Breast cancer, Colorectal cancer

## Abstract

**Background:**

Breast and colorectal cancer are the two most common cancers in Malaysia. Low awareness coupled with stigma and erroneous beliefs delay help-seeking behaviours, lead to late presentation and contribute to poor detection rates. Promoting cancer awareness through mass media may be effective in improving cancer-related knowledge and uptake in screening tests. However, research is sparse regarding the cultural translation and implementation of mass media campaigns in Malaysia (and Asia) in terms of raising awareness about colorectal and breast cancer.

**Methods:**

A collaborative partnership comprising researchers from Malaysia and the UK as well as policy makers, public health experts and non-government organisations from Malaysia was formed to design, deliver and evaluate the *Be Cancer Alert Campaign.* Each awareness-raising campaign will run for five weeks (Colorectal Cancer in April 2018, followed by Breast Cancer in October 2018). Evaluation of the campaigns will take place in Gombak district (Colorectal Cancer) and Petaling district (Breast Cancer) respectively, in the form of a pre-post randomly selected household survey and collection of service utilisation data. Occupants who are aged 40-years and above and are able to answer questions independently will be selected from each household. A sample of 730 with 80% power will detect a change of 6.09% in knowledge that unexplained lump or swelling is a symptom of breast cancer or changes in bowel habits is a symptom of colorectal cancer.

**Discussion:**

Malaysia and most South-East Asian countries have a low middle-income economy, with limited resources for cancer control. Late-staged cancers impose a significant economic burden on patients, households, communities, employers, health systems and governments. Our proposed strategy for the implementation of the culturally sensitive mass media cancer awareness-raising campaign will serve as a blueprint for cancer prevention and control policy in South-East Asian countries where the burden of cancer is increasing and there are high cancer death rates.

## Background

The global burden of cancer is increasing due to an ageing populations and the adoption of unhealthy life style behaviours in societies across the world. According to the GLOBOCAN 2008 report, the majority of cancer cases (56%) and cancer-related deaths (63%) occurred in countries with developing economies [1]. Cancer burden in Malaysia is predicted to rise and it is estimated that 1 in 9 females and 1 in 10 males will develop cancer before 75 years old [2]. Between 2007 and 2011, there were 103,507 newly diagnosed cancer cases and 64,275 cancer-related deaths [2]. It is estimated that about one-third to one-half of premature deaths due to cancer could be avoided through early presentation, detection and appropriate treatment [3]. However, cancers in Malaysia often present at late stages [4, 5] and it is likely that late presentation is due, at least partly, to low cancer awareness. For example, research indicates that there is a lack of awareness among Malaysian women about common symptoms of breast cancer (BC) [6, 7]. Similarly, knowledge about the warning signs for the most prevalent cancer in men, colorectal cancer (CRC) is poor [8, 9], i.e. only 40.6% of 2379 participants recognised bloody stool as a warning sign for CRC [8] and only 32.2% out of 384 women recognised ‘painless breast lump’ as a sign for BC [7]. Other causes of delayed detection and diagnosis include denial, negative perceptions of the disease, the over-reliance on traditional medicine, misperceived risk, emotional barriers and negative perceptions towards screening [10–12].

Advanced cancer impacts negatively on survival, requires more intensive and aggressive treatment, increases health care resource utilization and places additional financial burden on households [13]. Furthermore, studies in Malaysia demonstrate that there is socio-economic inequality regarding stage of presentation of cancer as well as survival rates. Kong et al. found that CRC patients from the lowest socio-economic group were significantly more likely to be diagnosed late with an advanced stage of the disease compared to patients from the highest socio-economic group [14]. In addition, significant differences in cancer awareness have been reported between rural and urban populations. Kanaga et al. reported that a rural population had significantly less knowledge about BC [15] and similar results have been reported for CRC [16]. Also, differences in knowledge have been found between the three main ethnicities in Malaysia (Malay, Chinese and Indian) [17]. Collectively, these findings demonstrate the need for cancer awareness raising programmes in Malaysia and that the programmes should take into account population demographic, socioeconomic and cultural variation.

In high income countries, nationwide public health interventions in the form of cancer awareness raising mass media campaigns have been developed and implemented in order to improve early presentation and diagnosis, and improve survival rates. For example, the *‘Screen for Life: National Colorectal Cancer Action Campaign*’ was launched in 1999 by the U.S. Department of Health and Human Services [18], in England the ‘*Be Clear on Cancer*’ *(BCOC)* programme was launched in 2011 [19] and, more recently in 2014, the ‘*Be Cancer Aware Programme*’ *(BCA)* was implemented by Northern Ireland’s Public Health Agency [20]. Evaluation findings from these campaigns reported an increased awareness about the signs and symptoms of cancer [21] (Public Health Agency. Evaluation Report Breast Campaign, unpublished) as well as increased attendance at GP practices by patients who reported key campaign-related symptoms [22]. In addition, more people in implementation areas were referred urgently by GPs for suspected cancer, there was an increase in diagnostic investigation activity, and a shift towards earlier stage diagnosis [23].

However, in countries comparatively less developed or with limited resources such as Malaysia mass-media campaign efforts have been sporadic, short-lived and lacked rigorous evaluation [24, 25]. There is a need to design a research-informed and culture-sensitive programme to improve awareness about cancer warning signs and to encourage early detection, downstaging of the disease and better treatment outcomes [26–28].

### Aims

Our main aim is to develop and evaluate a mass media campaign to raise awareness about Colorectal and Breast Cancer that is theoretically informed, evidence-based and culturally appropriate for Malaysia.

The specific objectives are to:

1. Develop a culturally-sensitive cancer awareness raising programme for Malaysia and organise its implementation
2. Design the evaluation framework and identify the evaluation metrics
3. Test the appropriateness, acceptability and feasibility of the programme.
4. Evaluate the impact of the programme to increase knowledge of signs and symptoms of BC and CRC as well as attitudes and beliefs about the two cancers.

The primary hypothesis is that promoting public awareness of cancer warning signs and symptoms and stressing the value of early presentation will result in down staging of cancer, an increased likelihood of receiving potentially curative treatment for patients leading to improved survival rates and better quality of life.

## Methods and design

### Promoting awareness of Cancer and early detection initiative (PACED)

The PACED initiative was instigated via a joint research call from the UK Medical Research Council-Newton Ungku Omar Fund for collaborative proposals from researchers in Malaysia and UK universities. A successful grant application from a research team led by Donnelly as the UK PI and Su as the PI in Malaysia led, in turn, to the development of a partnership between the University of Malaya, Queen’s University Belfast, the Ministry of Health Malaysia and the National Cancer Society Malaysia. This multidisciplinary partnership including researchers, policy makers, public health experts and non-government organisations (NGOs) in Malaysia is a forum for combining expertise in order to improve early presentation and diagnosis and improve long-term health outcomes for the general population. This paper presents the protocol for first project of the PACED initiative in Malaysia. The results will be used to shape and inform the rollout of future countrywide initiatives in Malaysia and other South-East-Asian countries.

### Campaign development

#### Cultural adaptation

The first project of the PACED initiative consists of two mass media cancer awareness raising campaigns under the slogan *‘Be Cancer Alert Campaign’* (BCAC). BCAC is based on successful programmes in other countries, principally the BCA campaign in Northern Ireland (Public Health Agency. Evaluation Report Breast Campaign, unpublished). BCAC will focus on the most prevalent cancers in Malaysia - BC and CRC. We are in the process of translating and adapting the BCA campaign, to ensure they are culturally sensitive towards the norms and beliefs of the new context and thus maximise the acceptability and reach of the campaign messages [29]. Several frameworks and guidelines exist for the cultural adaptation of health initiatives targeting minority populations e.g. the Chinese community living in the United States [29, 30] as well as for health initiatives adapted for use in different countries [31–33]. We have scoped the different frameworks and guides and evolved an integrated model of cultural adaptation that will be used to sensitise the programme to the country-context and population profile of Malaysia. A separate manuscript reporting the cultural adaptation of BCAC will be prepared in due course.

#### Evidence synthesis

We have completed two systematic reviews of the scientific literature regarding I) studies that assessed general population awareness and knowledge about, respectively, breast and colorectal cancer signs and symptoms and barriers towards screening in Malaysia and II) studies of mass and small media cancer campaigns in Asia that attempted to increase cancer awareness, knowledge and screening uptake for all cancers. Furthermore, a policy review is underway to describe the positioning of the campaign within the policy setting in Malaysia. The results of these reviews will be used to inform further the translation and implementation aspects of the programme including the content of the campaign, campaign messages and delivery.

#### Intervention design

**Fig. 1:**
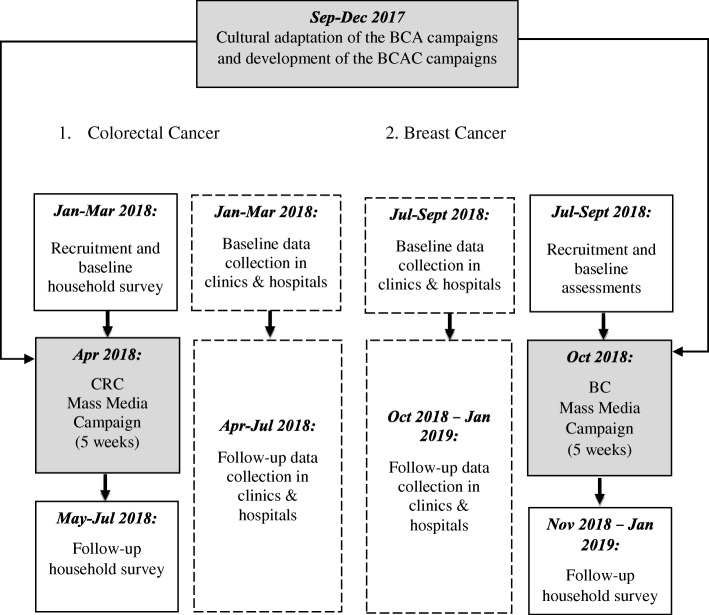
Timeline of the *Be Cancer Alert* campaigns

A population-based, intervention study will be piloted to identify the impact of the BCAC campaign to raise awareness about CRC and BC warning signs and symptoms. The CRC and BC campaign will be carried out and evaluated consecutively (Fig. 1).

### Setting

Malaysia has a multi-cultural population of around 28.7 million citizens, i.e. 68.8% of the population is Bumiputera (including Malay and other indigenous South-East Asians), 23.2% is Chinese and 7.0% is Indian [34]. The various communities follow their own religious beliefs and traditions, mainly Islam, Buddhism, Hinduism and Christianity. The national language is Bahasa Malayu, and many speak English. Other commonly spoken languages are Mandarin and Tamil. The majority of the population is aged between 15 and 64 years (69.7%) with a mean age of 28.3 years [34]. Malaysia has dual-tiered health care system with the public sector providing about 82% of inpatient care and 35% of ambulatory care and the private sector providing about 18% of inpatient care and 62% of ambulatory care  [35]. Malaysia does not have a national population-based screening programme for breast or colorectal cancer and instead cancer cases are diagnosed during opportunistic screening, requiring a patient to notice cancer symptoms or for a health care professional to suggest screening [36].

The campaigns will be piloted in Selangor state which is one of the most populated states in Malaysia (Population: 5,126,200, in 2011) [2]. The CRC campaign will be conducted in Rawang, a sub-district in Gombak and the BC campaign will be conducted in Sungai Buloh and Petaling Jaya, sub-districts in Petaling. According to the government Department of Statistics Malaysia (DOSM), each sub-district contains a mix of ethnicities, age groups and income groups and, therefore, they are representative of the general population of Selangor state.

### Study population

The target population are adults aged 40 years and above. This age-range has been selected as men and women in Low-Middle-Income Countries (LMIC) such as Malaysia are likely to be diagnosed at a younger age with BC and CRC [2] compared to high income countries which have observed a rapid increase in cancer diagnosis among adults aged 50 years old and above [37]. We will include I) men and women in the CRC campaign evaluation and women only for the BC campaign evaluation who are II) aged 40 years or older, III) speak English, and/ or Malay, IV) live in the randomly selected households from chosen study areas, V) and are able to provide answers independently without support from other adults.

Clinic data will be collected about colorectal and breast cancer-related outcomes in primary care clinics and hospitals (government and private) that are used by residents of the sampled areas from Gombak and Petaling, respectively. Special clinics, such as maternal and child health clinics will be excluded from the data collection.

### Sampling procedure

Random sampling will be applied to select the study population from each study area before the campaigns. Due to the nationwide-reach of some of the campaign materials via TV, radio and newspapers, it is not possible to construct meaningful comparator or ‘control’ sites. The same individuals will be interviewed before and after each campaign. A list of randomly selected households will be obtained from the DOSM. To obtain a list with 4000 households in each campaign area (oversampling to take into account non-respondents and participants not being eligible for survey) the DOSM will draw randomly 250 Enumeration Blocks (EBs) - artificially created contiguous geographical areas with specific boundaries containing 100 households. From each EB, 16 households will be selected randomly for inclusion in the household survey.

### Sample size

A sample of 730 participants in a pre-intervention survey of which 75% (550) participate in a post intervention survey will allow observation of a net change in awareness of unexplained lump or swelling as a symptom of breast cancer or changes in bowel habits as a symptom of colorectal cancer of 6.09% using the two sided McNemar Test for discordant proportions with alpha set at 0.05 and power at 80%. This assumes a negative shift in response among 10% of participants from the pre-intervention survey to the post-intervention survey and a corresponding 16.09% positive shift in response. A negative shift refers to participants who report awareness of the symptom before the intervention but lack of awareness following the intervention. Inevitably, some proportion of participants will report such a response. These are subtracted from those reporting a positive shift to yield a net change. We have no previous studies on which to base this assumption. A lower negative shift in response will allow observation of a smaller net change. For example, with a negative shift of 5%, the same sample size will allow observation of a net change of 4.55% while a 1% negative shift will allow observation of a net change of 2.54%.

### Intervention

#### Mass media campaign

The CRC and BC campaign will last for 5 weeks each and will be kick-started with a media launch at the University of Malaya in the first week of the campaigns to increase press coverage of the campaign. Similar to the BCA campaign, the key messages will be promoted through various media channels. This is in line with findings from a systematic review by Wakefield et al. who concluded that mass media interventions that utilise multiple channels to convey a message increase the likelihood of achieving successful positive impact [38]. The main delivery mode will be via TV advertisements since findings from previous national and international campaigns suggest that TV was the intervention mode with the highest reach [39, 40]. In addition, BCA evaluation findings in Northern Ireland suggested that posters in health care settings were the most commonly reported location (Public Health Agency. Evaluation Report Breast Campaign, unpublished) [20]. Therefore, a local (print) campaign will be implemented. In addition, radio and social media advertisements will be created as well as a study website. All materials will include the call to action to I) visit the campaign website for further information and to II) call the National Cancer Society toll-free helpline to discuss any questions.

#### Behaviour change theory and logic model

**Fig. 2:**
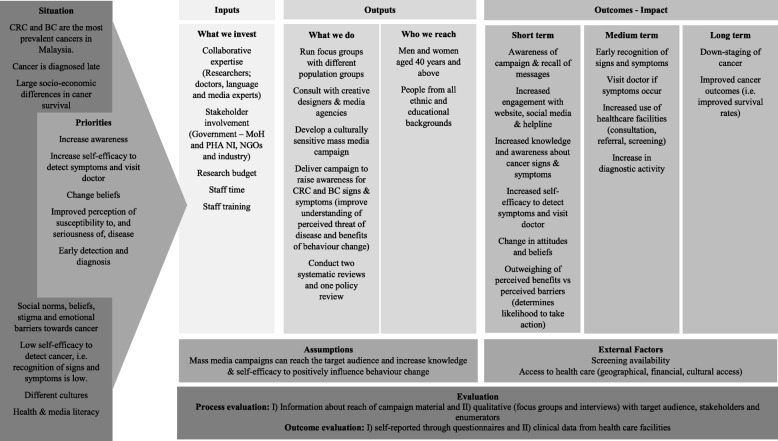
Logic Model for the *Be Cancer Alert Campaign***.** The *Be Cancer Alert Campaign* was instigated due to the high prevalence of CRC and BC and has been adapted to the current situation in Malaysia including the diverse population and socio-demographic differences. Informed by the success of the *Be Cancer Aware* campaign in Northern Ireland and the theoretical basis of the *Health Belief Model,* our priorities are to improve the perception of the susceptibility to, and seriousness of, cancer and to address the low awareness of signs and symptoms, particularly among people aged 40 and above. Close links with policy makers and NGOs increase the likelihood that the campaign is in line with government policy, well-informed and sustainable. Expected outcomes are an increase in uptake of screening and diagnostic activity due to improved knowledge and self-efficacy to detect early symptoms of cancer. Process and outcome evaluation in the form of self-reported as well as objective-measures will be used to demonstrate the impact of the campaign

The BCAC campaigns are guided by best available evidence, qualitative ‘testing’ and behavioural theory particularly the *Health Belief Model* (*HBM*) [41, 42]^,49^ which is one of the most commonly applied theories in cancer awareness raising programmes [43–45]. The *HBM* was developed in the 1950s by Hochbaum, Rosenstock and Kegels and comprises six main components: (1) perceived susceptibility, (2) perceived severity, (3) perceived benefits, (4) perceived barriers, (5) cues to action and (6) self-efficacy [42]. Individuals who perceive themselves to be susceptible to contracting a certain condition such as BC or CRC, (due for example to age, family history, or health behaviours) are more likely to perceive this condition as a threat. Similarly, people who perceive the condition in severe terms e.g. debilitating poor health are also more likely to view cancer as a threat. Individuals are more likely to develop self-efficacy to self-detect early warning signs and to visit a doctor if the perceived benefits of taking the necessary steps to improve health outcomes (e.g. attending screening to discover cancer early and treat it) outweigh the perceived barriers (e.g. negative perceptions towards screening). The likelihood of taking action (e.g. consulting a doctor and attending screening) is high if perceived threat and perceived self-efficacy are high. Continuous reminders through mass media adverts (TV, radio, print and online) will serve as repeated cues to action and remind individuals to see their doctor if necessary. Although, the use of a behaviour change theory is included in a number of studies, many studies do not describe the application of a logic model, including the BCA and BCOC campaigns in the UK. A recent Cochrane systematic review which published a logic model that explained the underlying theory for changing health behaviours in ethnic minority communities [46] highlighted the importance of understanding the target population, their context and situation and then using this understanding to designe tailored and targeted campaign messages. The logic model of the BCAC illustrates the theoretical guidance from the *HBM* (see Fig. 2).

### Outcome measures

The logic model presents the range of outcomes that the evaluation will attempt to capture. Our primary measure is a change in knowledge about campaign-related cancer symptoms (Fig. 2). In addition to increased awarareness about campaign materials and messages and engagement with the website, social media and the helpline, secondary outcomes include positive change relating to:

- knowledge between population groups from different ethnic (Malays, Chinese and Indian) and educational backgrounds.
- self-efficacy to detetct symptoms and visit doctor
- attitudes and beliefs about cancer
- number of patients discussing colorectal and breast cancer-related signs and symptoms with their doctor/ nurse (respectively)
- number of colorectal and breast cancer screening referrals and tests undertaken (respectively)
- number of colorectal and breast cancers diagnosed (respectively)

Secondary outcomes related to the campaign evaluation also include participant’s awareness about the campaign, recall of campaign messages and engagement with the campaign website, social media postings and utilisation of the helpline.

### Evaluation framework

We designed a comprehensive evaluation framework appropriate for the Malaysian population, infrastructure and healthcare system. It has been informed by the approach and methods used in the appraisal of the BCA campaign.

#### Methods of assessment and outcome measurement

##### Questionnaires

The household survey comprises a number of validated questionnaires that have been adapted for the Malaysian context previously or will be adapted and validated as part of this study. Socio-demographic (e.g. gender, ethnicity, religion, education, income, marital status, etc.) and health literacy information will be captured at baseline. Health literacy will be assessed with a validated 6-item scale (Mohamad E, Su TT, Pelikan J, Brouck S Van den, Sorenson K, Majid HA, et al. The validity and reliability of two short versions of the Health Literacy EU Survey (HLS-EU 16 items and HLS-EU 6 items) in Malaysia, unpublished) which is a shorter version of a validated 47-item scale  [47] to understand how people *‘access, understand, appraise, and apply health information in order to make judgments and take decisions in everyday life concerning healthcare, disease prevention and health promotion’* [48]. Findings may help us understand the change in knowledge and awareness as a result of the campaign [46].

In order to ascertain changes in cancer-related knowledge and awareness we are going to include the validated *C*ancer *A*wareness *M*easure (CAM) [16] before-and-after the implementation of each campaign. The Bowel CAM has been culturally adapted and validated previously for the Malaysian context from its original version developed in the UK [49] and consists of 24 items. The questions concern warning signs, barriers to seeking help, risk factors, cancer and age and confidence to notice cancer symptoms. We will adapt and validate the original version of the Breast Cancer CAM [50] for the purpose of the Breast Cancer BCAC. The validated *A*wareness and *B*eliefs about *C*ancer (ABC) measure [16] will assess change in attitudes and beliefs about cancer. Only questions that are not included in CAM and are relevant to the specific cancer (CRC and BC) will be included in the survey, i.e. items on awareness of cancer outcomes, help-seeking intentions, beliefs about cancer and about barriers to symptomatic presentation, and estimated age at which people are most likely to develop cancer.

In addition, we will use the *C*hampion *H*ealth *B*elief *M*odel *S*cale (CHBMS) to assess whether health beliefs linked to the underlying theory of the campaign change. The original CHBMS has been modified [51] and includes items that assess the following domains on a 5-point Likert Scale (strongly agree to strongly disagree): ‘perceived susceptibility’, ‘perceived barriers’ and ‘perceived benefits’ of screening. Also, it has been used in various settings, including Malaysia, to assess health beliefs with regards to breast self-examination and mammography [52–55]. We will investigate the validity of the CHMBS [51] for general breast cancer screening and colorectal cancer screening in Malaysia.

Lastly, at follow-up, we will include specific questions in the post-study questionnaire to appraise the extent to which the BCAC campaign messages were able to ‘cut through the noise’ and the public was able to recognise BCAC over other campaigns. Questions will be adapted from the BCA campaign, for example ‘*Which of the following were the main messages of what you recently saw, heard or read about cancer?*’, ‘*What slogans or phrases do you recall?*’ as well as direct questions about the campaign material, such as ‘*Have you seen this TV advertisement?*’

##### Engagement with website, social media and helpline

Findings from the BCA campaign suggest that website views peak close to the start of each campaign (i.e. over 8000 views at the start of the BC campaign) and decline soon after (Public Health Agency. Evaluation Report Breast Campaign, unpublished) [20]. Therefore, a marketing firm has been hired to monitor engagement with the website and social media. In addition, nurses who staff the help lines at the National Cancer Society Malaysia will monitor the number of people who called due to noticing the campaign advertisements as well as where people noticed the campaign and reason for calling. Callers who agree to the storage of their personal information will be followed up with a short-survey post-campaign to elicit their views about the campaign materials.

##### Clinical outcomes

Although the Malaysian Cancer Registry was established in 1997, the registration of cancer cases is voluntary and it does not appear to capture all cancer cases. Therefore, we have developed a pro-forma for local health care professionals from private clinics to capture I) the rate of before-and-after attendance at primary care physicians with BCAC symptoms, II) referral of patients to cancer screening, III) diagnostic tests undertaken and IV) cancer diagnosis. Data from government clinics will be collected retrospectively. Information will be collected from between one to three months pre-campaign, during the campaign and up to three months post-campaign to capture the screening results of all referred patients.

#### Data collection

Trained, bilingual research assistants will visit the randomly selected households and all eligible individuals in a household will be informed about the purpose of the survey and asked to participate. Written informed consent will be collected from all study participants. The research assistants will offer to carry out the interview in either English or Malay, face-to-face with each participant individually, without the influence of other family members. We will conduct two surveys (starting three months before and continuing until three months after each campaign, excluding the campaign period) (Fig. 1). Online engagement and clinical data will be collected as described earlier.

### Statistical analysis

Data from the questionnaires will be analysed with SPSS vs 24.0. Descriptive statistics at baseline will be reported as mean (SD) for continuous data and frequencies (percentages) for categorical data. Analysis of pre- and post-intervention data will be analysed with paired samples t-test for continuous variables and Pearson’s Chi-square for categorical variables. Multivariate regression models will be used to investigate variable influences on measures of cancer awareness and knowledge and change including influences relating to variables such as ethnicity and educational group (and any between-group differences).

## Discussion

In Malaysia, NGOs and industry are promoting cancer awareness through various media channels, particularly during cancer awareness months (e.g. October is BC awareness month) which often lasts between one day to one week [24]. Campaigns, so far, have focused mainly on BC and a small number of campaigns also focused on CRC [24] despite the fact that CRC is the most common cancer in Malaysian males. No formal evaluation of these campaigns were conducted. Therefore, the development of a theoretically informed, evidence-based and culturally appropriate cancer awareness-raising programme for Malaysia will add new insights regarding the approaches and methods of adapting, implementing and spreading successful health promotion programmes in Malaysia, South-East Asia and elsewhere. One of the main strength of the BCAC is the close engagement with policy makers and local NGOs, i.e. the Ministry of Health Malaysia and the National Cancer Society Malaysia. Also, our efforts to advance the rigour of the evaluation framework for the campaign and dovetail it to suit the local infrastructure and health care system will be beneficial to academics and change agents who engage in health promotion and public health interventions.

Robust scientific evidence regarding cancer awareness raising campaigns in Malaysia is sparse or non-existent. In 2010, an oral cancer awareness raising campaign, funded by a private hospital in Malaysia was promoted through TV and radio advertisement as well as a talk show at the end of a 32-day campaign. Although the number of people who heard of oral cancer increased, level of awareness about signs and symptoms remained unchanged [40]. Two separate RCTs [56, 57] found that small media campaigns (i.e. letters, telephone calls and/or text messages) increased pap smear uptake in Malaysia. UK mass media campaigns promoting mammography appeared to increase uptake when screening facilities were in place [38]. Similar findings were reported for CRC referral [58]. It is not clear whether or not a similar outcome would pertain in a LMIC such as Malaysia.

Our campaign will address key objectives of the Ministry of Health’s (MoH) *‘National Strategic Plan for Cancer Control Programme 2016-2020’* including the stated need for a study of the kind presented here. The strategic objectives include: I) increasing screening coverage for targeted populations (educate public about the importance of cancer screening); II) increasing awareness about early warning signs and symptoms in the population and among health care providers; III) assessing awareness and knowledge among the general public regarding common cancers in Malaysia; and IV) strengthening cancer research by recruiting and keeping talent as well as combining efforts from the public sector and research in universities [59].

The burden of cancer in South-East Asian countries is increasing. As most ASEAN countries have low or middle-income economies, there are limited resources for cancer control, especially early cancer detection and cancer treatment, which may explain higher cancer death rates in this region. Late staged cancers impose a significant biopsychosocial burden for patients and families and an economic and social burden for communities, employers, health systems, and governments. Our programme development, implementation and evaluation of cancer awareness raising will serve as a blue print for cancer prevention and control policy in ASEAN where the burden of cancer is increasing and there are high cancer death rates.

## Abbreviations

ABC: Awareness and Beliefs about Cancer (evaluation tool)
BC: Breast Cancer
BCA: Be Cancer Aware Campaign
BCAC: Be Cancer Alert Campaign
CAM: Cancer Awareness Measure (evaluation tool)
CHBMS: Champion Health Belief Model Scale (evaluation tool)
CRC: Colorectal Cancer
DOSM: Department of Statistics Malaysia
HBM: Health Belief Model
LMIC: Low Middle Income Countries
MOH: Ministry of Health
